# Editorial: The Role of Neuropeptides in Drug Addiction and Other Psychiatric Disorders

**DOI:** 10.3389/fnbeh.2022.952551

**Published:** 2022-06-28

**Authors:** Kabirullah Lutfy, Lucia Hipolito, Valentina Ferretti, Leandro F. Vendruscolo, Marsida Kallupi

**Affiliations:** ^1^College of Pharmacy, Western University of Health Sciences, Pomona, CA, United States; ^2^Department of Pharmacy and Pharmaceutical Technology and Parasitology, University of Valencia, Burjassot, Spain; ^3^Center for Research in Neurobiology Daniel Bovet, Sapienza University of Rome, Rome, Italy; ^4^Integrative Neuroscience Research Branch, National Institute on Drug Abuse, Intramural Research Program, National Institutes of Health, Baltimore, MD, United States; ^5^Department of Psychiatry, San Diego School of Medicine, University of California, San Diego, San Diego, CA, United States

**Keywords:** addiction, glucagon, leptin, orexin, PACAP, MDMA, corticotropin releasing factor (CRF), dynorphin

This Research Topic was aimed to publish breakthrough findings encompassing the role of neuropeptides in addiction and other neuropsychiatric disorders. The data collected here, explain the involvement of neuropeptides in addiction-like behaviors both at the cellular and at the systemic level.

Cocaine addiction represents a major public health issue and negatively impacts society and the economy. However, there is no pharmacotherapy currently available to treat this chronic and relapsing brain disorder. Recent evidence suggests that peptides, such as glucagon-like peptide-1 (GLP-1) and leptin, which are involved in satiety and glucose and energy homeostasis, may also be functionally involved in cocaine reward and reinforcement. Zhu et al. used the place conditioning paradigm to study the effect of exendin-4, a GLP-1 agonist, on the reinstatement of cocaine-induced conditioned place preference, a model of drug reward. Reinstatement was elicited by either a priming dose of cocaine (10 mg/kg) or exposure to stress; drug and stress exposure can lead to relapse in people with cocaine use disorder. Exendin-4, given 1 h before each extinction training, attenuated reinstatement of cocaine- or stress-induced conditioned place preference. Additionally, Western Blot analysis was used to assess changes in the level of a nuclear transcription factor, NF- κB, in the nucleus accumbens in response to cocaine and stress exposure. Treatment with Exendin-4 also reduced NF-κB levels in the nucleus accumbens.

Glucagon-like peptide 1 receptors (GLP-1Rs) are highly expressed in the brain and are responsible for mediating the acute anorexigenic actions of GLP-1R agonists. In another multidisciplinary study, Zeng et al. performed a deep anatomical and neurophysiological characterization of GLP-1Rs in the central nucleus of the amygdala (CeA), where they found that GLP-1R is diffusely coexpressed in known CeA neuronal subpopulations like protein kinase, somatostatin, and tachykinin. They next mapped the anatomical positions of the GLP-1R-containing cells by using Glp1r-Cre mice and viral Cre-dependent tracing. They found that Glp1r-CeA cells are highly enriched in the medial subdivision of the CeA. By electrophysiological whole-cell recordings, the authors found that Glp1r-CeA neurons are characterized by the presence of hyperpolarized activated inward (Ih)-like currents, with a sex difference in magnitude and membrane capacitance. Future studies leveraging these data will be important to understanding the impact of GLP-1R agonists on reward and motivation, and may identify potential targets for developing pharmacotherapy to treat disorders associated with cocaine and food intake.

In this Research Topic, a review article by Keller et al. analyzed the involvement of GLP-1 in the reward system as one of the neuropeptides that contributes to the homeostatic control system, which is often disrupted by alcohol consumption and withdrawal.

In another study, Carrette et al. examined the potential relationship between leptin and cocaine self-administration in genetically heterogeneous rats. The authors processed 120 blood samples from 60 rats. They did not find a relationship between the body weight of animals and cocaine self-administration during withdrawal and abstinence. However, baseline blood leptin levels could predict addiction-like behaviors. They observed that the higher the basal leptin levels, the lower the cocaine self-administration after cocaine withdrawal and abstinence. The authors further confirmed this finding in a separate cohort of rats, showing that leptin administration reduced cocaine seeking during cocaine protracted abstinence. Together, these results suggest that peripheral peptides involved in glucose and energy homeostasis may have a protective effect against cocaine seeking and craving.

Another neuropeptide, pituitary adenylate cyclase activating polypeptide (PACAP), implicated in homeostatic systems within the body, has been recently found to be involved in substance use disorder, specifically in alcohol use disorder. Minnig et al. investigated the potential contribution of the PAC1 receptor in the nucleus accumbens shell in alcohol drinking in rats using a viral vector-mediated knockdown approach. By using operant models of oral self-administration in rats, the authors found that the loss of function of PAC1 receptor in the nucleus accumbens shell leads to increased alcohol drinking and increased motivation for alcohol, suggesting that the PACAP/PAC1 receptor system in this brain area may act as a “brake” on excessive alcohol drinking.

Among other neuropeptides that play a functional role in motivated behaviors, β-endorphins have been implicated in cocaine reward. In this work, Singh and Lutfy investigated the role of β-endorphins in cocaine-induced conditioned place preference, extinction, and reinstatement in male and female mice lacking β-endorphin and their wild-type controls. The authors found that β-endorphin is involved in the rewarding actions of cocaine in both male and female mice. However, female mice lacking β-endorphin exhibited place preference following repeated conditioning with cocaine, whereas male mice did not. Nonetheless, re-exposure to cocaine failed to reinstate the place preference response in mice lacking β-endorphin regardless of sex. Future studies will be important to further investigate the involvement of β-endorphins in the escalation and relapse of psychostimulants in rodents.

The entactogen psychostimulant drugs, 3,4-methylenedioxymethcathinone (methylone), 3,4-methylenedioxypentedrone (pentylone), and 3,4-methylenedioxy methamphetamine (MDMA) are commonly used substances. In this Research Topic, Khom et al. investigated whether female rats escalate self-administration of methylone, pentylone, and MDMA; they then studied the consequences of MDMA and pentylone self-administration on GABA_A_ receptor and κ-opioid receptor signaling in the CeA. Within the class of entactogen stimulants, the propensity to have a stable self-administration pattern is variable. Importantly, to date, there are limited data available that elucidate the use liability of entactogen stimulants in female subjects, and that may be related to the limited exposure to the drug. Thus, the authors initially determined whether long access to intravenous self-administration of three entactogens leads to escalation of drug intake in female rats, as it does in males. The authors used the acquisition of self-administration under long-access conditions and post-acquisition dose substitutions under a progressive ratio schedule of reinforcement to assess potential differences in behavioral patterns in entactogen self-administration. Indeed, female rats readily acquired methylone, pentylone, and MDMA self-administration under long-access daily conditions. Lastly, they investigated the neuroadaptations in synaptic transmission in the CeA by performing whole-cell patch-clamp electrophysiology to assess changes in CeA GABA transmission and its regulation by the dynorphin/κ-opioid receptor system in female MDMA- and pentylone-exposed rats. They demonstrated a significant dysregulation of CeA neuronal activity in response to self-administration of entactogens in female rats, reinforcing the importance of performing behavioral and cellular studies in both sexes.

In a review published in this Research Topic, Cassello et al. summarized information regarding neuropeptide signaling in the prefrontal cortex (PFC) and its relevance to neuropsychiatric disorders. They reviewed data on dynorphin, enkephalin, corticotropin releasing factor (CRF), and some others. This area is an exciting emerging field of study with significant potential for clinical translation. The authors did an excellent job balancing the biological and clinical aspects of each neuropeptide, establishing knowns and unknowns, discussing the potential role of neuropeptides in neuropsychiatric disorders and animal models, and providing insights for future research.

In another review article, Matzeu and Martin-Fardon summarized the literature on the role of orexin/hypocretin neuropeptides on alcohol, opioid, nicotine, and cocaine addiction. Orexin receptor antagonists have been recognized among other potential medication targets for addiction treatment. However, it is unclear whether these compounds are equally effective across different drugs of addiction or in polysubstance use, and whether they are efficacious in specific stages of the addiction cycle. Also unknown is whether the efficacy of the orexin receptor antagonists could be sex-specific. Investigating the role of these compounds on additional models of substance use disorder and in clinical research will help elucidate how the orexin system contributes to neuropsychiatric disorders, and find novel treatments for these disorders.

Finally, Curley et al. provide an overview of recent studies regarding the molecular mechanisms related to the role of corticotropin releasing factor binding protein (CRFBP) in the progression of addiction and other psychiatric disorders, biological aging, and age-related neurodegenerative disease. Dysregulation of the CRF stress system is associated with psychiatric and neurodegenerative disorders, and CRFBP has been indicated as a novel target to mediate the contribution of stress-related dysfunction to these conditions. However, future studies on CRFBP should examine sex as a biological variable, as well as consider the relevance of ethnic background and genetic polymorphisms that may alter susceptibility to stress-related disorders.

## Author Contributions

All authors listed have made a substantial, direct, and intellectual contribution to the work and approved it for publication.

## Conflict of Interest

The authors declare that the research was conducted in the absence of any commercial or financial relationships that could be construed as a potential conflict of interest.

## Publisher's Note

All claims expressed in this article are solely those of the authors and do not necessarily represent those of their affiliated organizations, or those of the publisher, the editors and the reviewers. Any product that may be evaluated in this article, or claim that may be made by its manufacturer, is not guaranteed or endorsed by the publisher.

